# Postoperative outcomes of biopsy versus debulking surgery for immunoglobulin G4-related ophthalmic disease: a retrospective comparative study

**DOI:** 10.1007/s10384-024-01157-0

**Published:** 2025-01-20

**Authors:** Rikako Iwasaki, Yoshiyuki Kitaguchi, Takeshi Morimoto, Kohji Nishida

**Affiliations:** 1 Department of Ophthalmology, Osaka University Graduate School of Medicine, 2-2 Yamadaoka, Suita, 565-0871 Osaka Japan; 2 Division of Health Science, Department of Medical Physics and Engineering, Osaka University Graduate School of Medicine, Osaka, Japan; 3 Institute for Open and Transdisciplinary Research Initiatives (OTRI), Osaka University, Suita, Osaka Japan

**Keywords:** Biopsy, Debulking, IgG4-related ophthalmic disease, Treatment

## Abstract

**Purpose:**

To compare the postoperative outcomes of corticosteroid therapy following biopsy with those following debulking surgery in patients with immunoglobulin G4 (IgG4)-related ophthalmic disease (IgG4-ROD).

**Study Design:**

Retrospective comparative study.

**Methods:**

Fifteen patients diagnosed with IgG4-ROD (5 unilateral, 10 bilateral) were retrospectively analyzed. IgG4-ROD was diagnosed based on imaging, histopathology, and blood test results. The biopsy group included patients who underwent resection of minimal tissue for diagnosis, whereas the debulking group included patients who underwent resection of a substantial portion of the mass to decrease the tumor size. Postoperative outcomes after steroid administration, recurrence rates, and changes in lacrimal gland function were compared between the groups.

**Results:**

The biopsy and debulking groups included seven and eight patients, respectively. All patients in the biopsy group and 25% of patients in the debulking group required steroid treatment postoperatively (*p* = 0.0070). Relapse occurred in 71.4% and 12.5% (*p* = 0.041) and maintenance therapy was required in 57.1% and 12.5% (*p* = 0.12) patients in the biopsy and debulking groups, respectively. Twelve patients had extraorbital lesions, with one patient receiving corticosteroid treatment for sphenoid bone lesion. Schirmer I test values did not differ preoperatively and postoperatively in either group (biopsy: *p* = 0.47; debulking: *p* = 0.72). One patient from the biopsy group developed severe dry eyes, necessitating lacrimal canalicular excision.

**Conclusions:**

Debulking surgery effectively reduced the requirement for postoperative steroid administration for recurrent lacrimal gland lesion in patients with IgG4-ROD, indicating its potential as an effective alternative to current standard treatment.

## Introduction

Immunoglobulin G4 (IgG4)-related disease is an autoimmune condition characterized by fibrosis occurring in multiple organs because of infiltration of lymphocytes and IgG4-positive plasma cells [1]. Ophthalmic involvement is a manifestation of IgG4-related disease observed in 3.9–23% of cases [2–4] and presents with distinct symptoms, including eyelid swelling, proptosis, dry eye, diplopia, reduced visual acuity, and visual field defects [2, 3, 5]. These symptoms impact the quality of life and daily functioning of affected individuals, underscoring the importance of efficacious management strategies for IgG4-related ophthalmic disease (IgG4-ROD).

Currently, corticosteroid therapy is the primary treatment for IgG4-ROD after a definitive diagnosis has been made using biopsy [5–9]. Despite its effectiveness, corticosteroid treatment is associated with high recurrence rates and potential adverse systemic effects after extended use [3, 5, 7–9]. An alternative treatment, particularly in cases of IgG4-ROD, is debulking surgery; it shows a comparatively lower recurrence rate, suggesting its potential as an effective treatment modality [6, 10]. However, direct comparison of relapse rates, steroid dependence, and complications between biopsy and debulking is lacking.

This study aimed to compare the postoperative outcomes of corticosteroid therapy following biopsy and debulking surgery in patients with IgG4-ROD. By evaluating factors such as corticosteroid dependency, recurrence rates, and changes in lacrimal gland function, we aimed to identify the optimal management strategy for this condition.

## Materials and methods

### Study design

This was a retrospective chart review of Japanese patients diagnosed with IgG4-ROD at our university hospital between March 2017 and February 2022.

We first reviewed the patients’ medical records and identified patients diagnosed with definite IgG4-ROD using the following comprehensive diagnostic criteria based on imaging, histopathology, and blood test results: (1) imaging results indicating enlargement or lesions in the lacrimal gland, trigeminal nerve, extraocular muscle, or other ocular tissues; (2) histopathological findings of lymphocyte and plasma cell infiltration with an IgG4(+)/immunoglobulin G(+) cell ratio ≥ 40% or ≥ 50 IgG4(+) cells per high-power field; and (3) elevated serum IgG4 levels (≥ 135 mg/dl) [11]. Among the identified patients, those with primary symptoms associated with lacrimal gland enlargement, such as upper eyelid swelling and the presence of a palpable mass, were included in this study. Patients with a follow-up period of less than 1 year after incisional biopsy were excluded.

This study complied with the tenets of the Declaration of Helsinki. The Institutional Review Board of Osaka University Hospital approved this study (no. 23212) and waived the requirement for obtaining informed consent based on the ethical guidelines for medical and health research involving human subjects established by the Japanese Ministry of Education, Culture, Sports, Science, and Technology and the Ministry of Health, Labour and Welfare. However, an outline of the study was made available for public viewing on the university hospital website at the request of the institutional review board to give patients the opportunity to decline participation. None of the patients withdrew their participation. The medical records were anonymized prior to data analysis.

### Data collection

Data regarding the age, sex, follow-up period after surgery, presence or absence of IgG4-related systemic lesions, Schirmer I test values at the preoperative and final visits, serum IgG4 levels at the preoperative and final visits, lesion volume, extent of lesion excision, and postoperative outcomes were extracted from the medical records. We outlined the volume of the lesion in consecutive axial slices of computed tomography (CT) or magnetic resonance imaging (MRI) scans and calculated the volume. Specifically, the volumes of the palpebral lobe and the orbital lobe were measured separately.

**Fig. 1:**
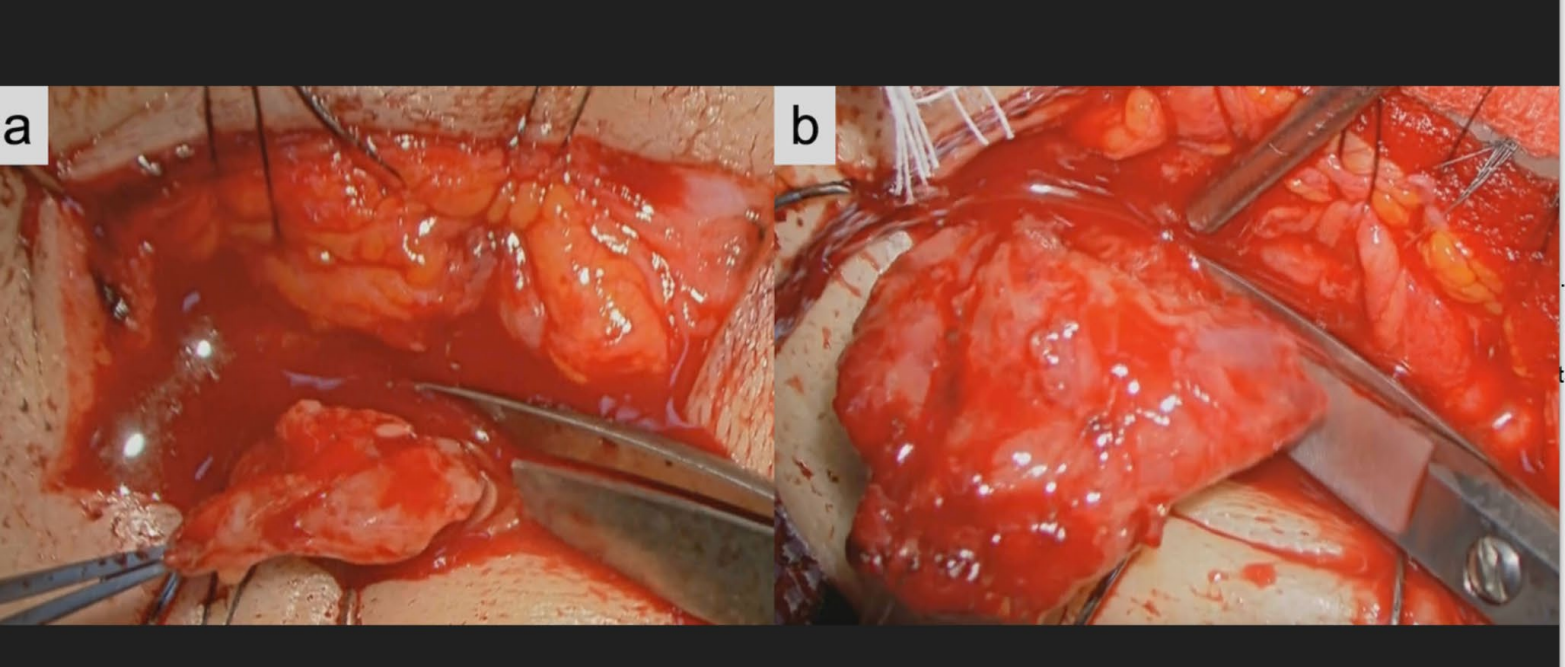
Intraoperative findings representing the extent of lesion excision from the surgeon’s perspective under a microscope. a The biopsy group. The specimen is divided into three pieces for histopathological examination, flow cytometry, and genetic rearrangement analysis. b The debulking group. The lesion is excised at the junction between the orbital and palpebral lobes of the lacrimal gland

Patients were categorized into either the biopsy or debulking group based on the extent of lesion excision. The extent of lesion excision was minimal in the biopsy group, and three specimens nearly the size of the tip of the little finger were collected for histopathological examination, flow cytometry, and gene rearrangement analysis (Fig. 1) [12, 13]. In contrast, most of the orbital lobe of the lacrimal gland was resected and the palpebral lobe was preserved in the debulking group to prevent severe dry eye [14]; resection was achieved by dissecting the lesion at the boundary between the palpebral and orbital lobes (Fig. 1). All surgery was performed via the eyelid crease approach, and specimens were collected from the orbital lobe. No corticosteroids were administered to any of the patients prior to the surgery or intraoperatively.

The choice of surgical technique—biopsy or debulking—was determined solely by the timing of surgical indication, not patient condition. Prior to June 2019, biopsy was performed in all cases, under either local or general anesthesia. However, a shift to a preference for debulking surgery occurred in June 2019, influenced by growing evidence demonstrating its benefits in reducing recurrence rates without functional complications [6]. Following this shift, debulking surgery became the preferred recommendation for all cases, typically performed under general anesthesia. Despite this shift, some patients opted for biopsy due to their preference for a minimally invasive procedure with local anesthesia. The surgical technique was selected without consideration of the patient’s preoperative symptoms, such as lacrimal volume and presence or absence of dry eyes.

The frequency of postoperative administration of oral corticosteroids was compared between the biopsy and debulking groups. At our hospital, the protocol for oral corticosteroids comprised the administration of 30 mg/day of prednisolone, with the dose tapered to 5 mg/day over 3 months. Steroid treatment was discontinued after 6 months to 1 year if the symptoms were resolved [7, 15, 16]. The recurrence of eyelid swelling or a palpable mass in the lacrimal gland area was defined as relapse [7, 15, 16]. For bilateral cases, recurrence in either or both lacrimal glands was considered as relapse. Patients with relapse were administered maintenance therapy of 5 mg/day of corticosteroids. Patients who remained symptomatic at a dose of 5 mg/day were designated as non-responders and were administered a maintenance dose of 5 or 10 mg/day of corticosteroids.

The outcome analysis also included changes in lacrimal gland function. The preoperative and postoperative Schirmer I test values and the use of eye drops for treating dry eyes were compared to evaluate the lacrimal gland function.

### Statistical analyses

Data are expressed as mean values with accompanying interquartile range (IQR). Mann-Whitney U test was used to compare age, follow-up period, Schirmer I test values, serum IgG4 levels, and lesion volumes between the two groups. Fisher’s exact test was used to compare sex, the proportion of patients with systemic lesions, corticosteroid administration, relapse rate, maintenance corticosteroid therapy administration, and eye drop administration between the two groups. The Wilcoxon signed-rank test was used to evaluate the changes in Schirmer I test values in each group preoperatively and postoperatively. Statistical significance was set at *p* < 0.05.

## Results

### Demographic characteristics

Fifteen patients (5 unilateral, 10 bilateral) with IgG4-ROD were enrolled in this study. Seven and eight patients underwent incisional biopsy and debulking surgery, respectively.

Table 1 presents the patient demographics. The mean age of the patients was 64 years (IQR: 55-72.5 years, range: 46–79 years). The study population comprised five men and ten women. The preoperative Schirmer I test values and serum IgG4 levels were 6.35 mm (IQR: 2–6 mm) and 981 mg/dL (IQR: 450–980 mg/dL), respectively. Twelve patients had IgG4-related systemic lesions (Table 2). The volumes of the measured lesions calculated using preoperative CT or MRI were as follows: total volume was 2467 mm³ (IQR: 1813–2827 mm^3^), palpebral lobe volume was 332 mm³ (IQR: 231–400 mm^3^), and orbital lobe volume was 2135 mm³ (IQR: 1326–2473 mm^3^) (Figure appendix 3–6). The percentage of orbital lobes in the total volume was 82.9% (IQR: 79.8–89.7%, range: 61.6–91.9%) in the biopsy group and 86.2% (IQR: 86.4–92.4%, range: 65.9–94.3%) in the debulking group, which was not significantly different (*p* = 0.17). Thus, the extent of lesion excision was estimated to be 70–90% of the total volume of the swollen lacrimal gland. No significant differences were observed between the two groups in terms of baseline clinical characteristics, including age, sex, preoperative Schirmer I test values, preoperative serum IgG4 levels, presence or absence of IgG4-related systemic lesions, and volume of the lesions.

**Table 1:** Demographic characteristics of the patients

Characteristic	Biopsy group (*n* = 7)	Debulking group (*n* = 8)	*p* value
Age, years (mean (IQR), range)	69.4 (68.5–74.5), 49–79	59.3 (52-65.3), 46–76	0.092^*^
Sex, male: female	3:4	2:6	0.61^†^
Preoperative Schirmer I test values, mm (mean (IQR), range)	10.2 (3.3–12), 0–30	3.38 (1–5), 0–7	0.11^*^
Preoperative serum IgG4 levels, mg/dL (mean (IQR), range)	773 (536–958), 218–1450	1228 (458–978), 212–4660	0.95^*^
Presence of IgG4-related systemic lesions, n (%)	5 (71.4)	7 (87.5)	0.57^†^
Volume of the lesion (total), mm^3^ (mean (IQR), range)	2170 (1510–2722), 1040–3655	2741 (2164–2827), 1370–5151	0.24^*^
Volume of the lesion (palpebral lobe), mm^3^ (mean (IQR), range)	326 (233–359), 218–630	337 (180–415), 135–618	0.85^*^
Volume of the lesion (orbital lobe), mm^3^ (mean (IQR), range)	1844 (1198–2490), 640–3025	2404 (1820–2473), 943–4588	0.29^*^
Percentage of the orbital lobe in the total volume, % (mean (IQR), range)	82.9 (79.8–89.7), 61.6–91.9	86.2 (86.4–92.4), 65.9–94.3	0.17^*^

*IgG4* immunoglobulin G4, *IQR* interquartile range

^*^Calculated using Mann-Whitney U test

^†^Calculated using Fisher’s exact test

**Table 2:** Systemic involvement of IgG4-related disease in the study population

Organ	No. (%) (*n* = 15)
Lung	6 (40)
Submandibular glands	5 (33)
Kidney	3 (20)
Respiratory tract	2 (13)
Mediastinum	2 (13)
Lymph nodes	2 (13)
Lateral rectus muscle	2 (13)
Retroperitoneum	1 (7)
Parotid gland	1 (7)
Pharynx	1 (7)
Sphenoid bone	1 (7)

### Comparison of postoperative outcomes

**Fig. 2:**
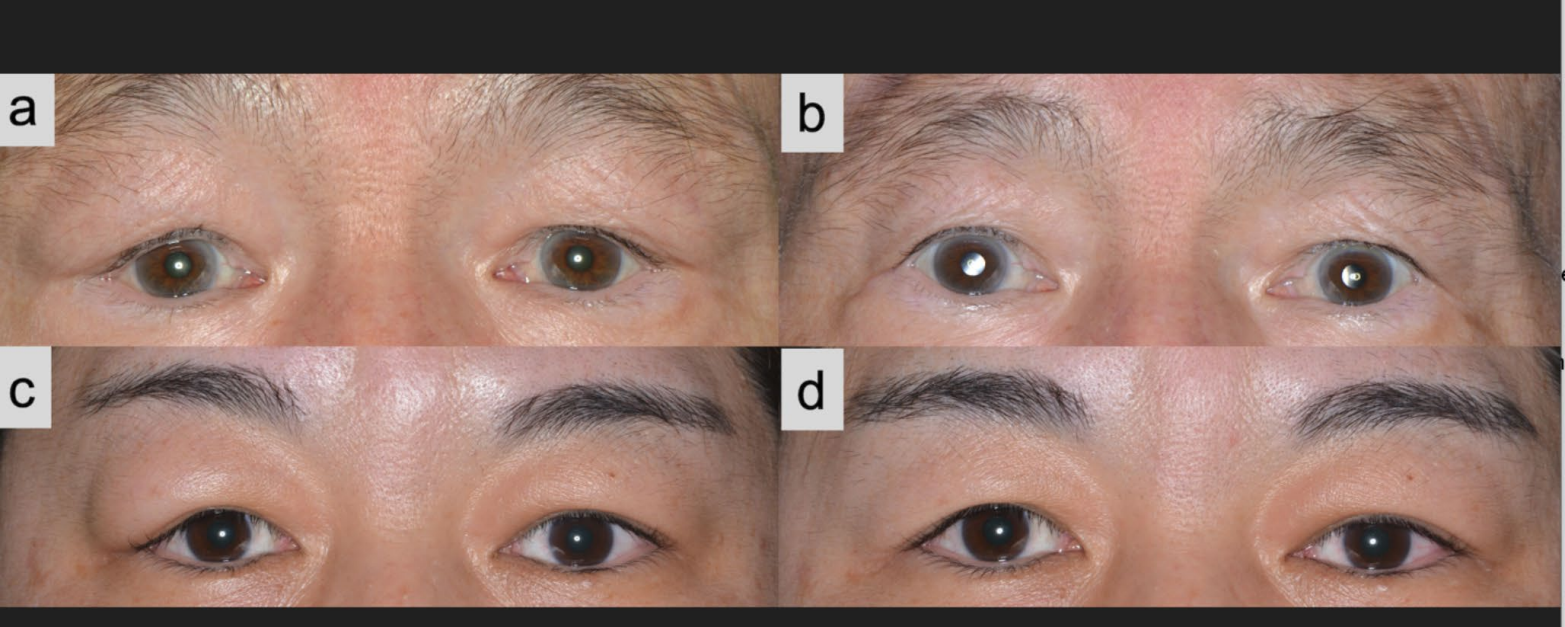
Photographs of the typical clinical course in patients who underwent biopsy or debulking surgery. a Preoperative and b postoperative images of a representative case in which the patient underwent lacrimal gland incisional biopsy and was treated with corticosteroids. c Preoperative and d postoperative images of a representative case in which the patient underwent debulking surgery without corticosteroid treatment

Table 3 presents the postoperative clinical outcomes of the two groups. The follow-up periods after surgery were 53.6 months (IQR: 47.5–58.5 months) and 31.8 months (IQR: 20.3–42 months) for the biopsy and debulking groups, respectively (*p* = 0.011). All seven (100%) patients in the biopsy group and two of the eight (25%) patients in the debulking group received oral corticosteroids following pathological diagnosis (*p* = 0.0070). Of note is that one patient in the debulking group was diagnosed with concurrent sphenoid bone involvement and was administered corticosteroids for the disease, indicating that only one patient required postoperative corticosteroid treatment for residual periocular swelling after surgery. Except for the patient with sphenoid lesions, no patients were administered corticosteroid treatment for systemic lesions. After treatment, all patients experienced an improvement in eyelid symptoms associated with lacrimal gland enlargement (Fig. 2). We have included with the present study pre-operative and post-operative CT or MRI images of all cases (Figure appendix 3–6). Although post-operative images were not available in several cases, no obvious recurrent swelling of the lacrimal glands was demonstrated in the debulking group.

**Table 3:** Postoperative clinical outcomes

Outcome	Biopsy group (*n* = 7)	Debulking group (*n* = 8)	*p* value
Follow-up period, months (mean (IQR), range)	53.6 (47.5–58.5), 40–68	31.8 (20.3–42), 12–48	0.011^*^
Corticosteroid administration, n (%)	7 (100)	2 (25)	0.0070^†^
Relapse, n (%)	5 (71.4)	1 (12.5)	0.041^†^
Maintenance corticosteroids, n (%)	4 (57.1)	1 (12.5)	0.12^†^
Schirmer I test values, mm (mean (IQR), range)	9.1 (5.5–13.8), 3–15	3.2 (1–6), 0–8	0.0022^*^
Serum IgG4 levels, mg/dL (mean (IQR), range)	316 (171–458), 74–510	944 (358–919), 102–3450	0.16^*^

IgG4 immunoglobulin G4, *IQR* interquartile range.

^*^Calculated using Mann-Whitney U test. ^†^Calculated using Fisher’s exact test

Postoperative relapse occurred in five (71.4%) patients in the biopsy group and one (12.5%) patient in the debulking group (*p* = 0.041); among them, one patient in the biopsy group opted for observation without corticosteroids. In all cases of recurrence in the debulking group, the recurrent symptom was not a palpable mass but eyelid swelling. Five of the 10 patients with bilateral disease experienced recurrence, with all five having bilateral recurrences. The time from surgery to relapse was 27.7 months. Consequently, four (57.1%) patients in the biopsy group and one (12.5%) patient in the debulking group (*p* = 0.12) received corticosteroid maintenance therapy.

Side effects of corticosteroid treatment were observed in two patients in the biopsy group. One patient was diagnosed with new-onset diabetes as a result of undergoing steroid treatment; the other had worsening of preexisting diabetes and required insulin therapy.

Schirmer I test values did not differ between pre- and postoperative measurements in either the biopsy group (*p* = 0.47) or the debulking group (*p* = 0.72). However, at the final follow-up, mean Schirmer I test values were significantly lower in the debulking group compared to the biopsy group (3.2 mm (IQR: 1–6 mm) vs. 9.1 mm (IQR: 5.5–13.8 mm), *p* = 0.0022). The number of patients who received eye drop treatment for dry eyes increased from two (25%) to six (75%) among the eight patients in the debulking group; however, this change was not statistically significant (*p* = 0.13; Table 4). Most patients in both groups did not report dry eyes or were managed effectively with one eye drop measure administered three to six times a day. One patient in the biopsy group developed severe dry eyes due to the natural progression of preexisting dry eyes and required punctal plugs and excision of the horizontal canaliculus, which was performed using a previously reported method [17].

**Table 4:** Changes in the rate of eye drop usage for dry eye disease among patients pre- and postoperatively

Group	Preoperative	Postoperative	*p* value
Biopsy group, n (%)	2 (28.6)	2 (28.6)	1.00^*^
Debulking group, n (%)	2 (25)	6 (75)	0.13^*^

^*^Calculated using Fisher’s exact test

## Discussion

In this study, a significant difference was demonstrated in the clinical outcomes between patients undergoing biopsy and those undergoing debulking surgery for IgG4-ROD. The debulking surgery group exhibited reduced corticosteroid dependency and lower recurrence rates compared with the biopsy group. These findings are consistent with previous studies that report recurrence rates of 36–75% following corticosteroid therapy and 13% following debulking surgery [6–8, 15, 16, 18, 19]. The results of our study support these figures, with recurrence rates of 12.5% in the debulking group and 71.4% in the biopsy group being observed. The uniqueness of our study is its direct comparison of these two treatment methods within the same clinical environment, an aspect not previously explored in the literature.

One recurrent case after debulking surgery presented with eyelid swelling without a palpable mass in the lacrimal gland area. This observation indicates that while debulking surgery effectively reduces lacrimal gland mass, it does not entirely suppress inflammation in the eyelid tissue. Previous studies report that lacrimal gland enlargement occurs in 68–88% of IgG4-ROD cases, while eyelid swelling is observed in 12–21% of cases [2, 5, 20]. While debulking surgery alone may be sufficient for most cases primarily involving the lacrimal gland, patients presenting with prominent eyelid swelling, a less common symptom, might require additional management strategies, such as steroid administration. Further studies with larger cohorts and longer follow-up periods are needed to better understand the frequency and characteristics of swelling recurrence after debulking surgery, as well as the rate of new-onset eyelid swelling during follow-up.

In this study, we administered a standardized dose of 30 mg/day of prednisolone to all patients, irrespective of their body weight. While, based on existing literature this dosage falls within the recommended range of 0.5–1 mg/kg/day [7, 15, 16], it is important to acknowledge that, due to variations in body weight a fixed dose does not yield equal efficacy among all patients. For example, a 30 mg dose might be relatively high for a patient weighing 30 kg but insufficient for one weighing 80 kg. This variation in effective dosages potentially influences treatment outcomes, including the rate of relapse and the need for maintenance therapy.

Reports indicate that 40–56% of patients develop new-onset diabetes or worsening of diabetes after undergoing oral corticosteroid therapy [21, 22]. In the present study, one individual developed diabetes and another experienced a worsening of his condition. Although both these patients were in the biopsy group, complications of diabetes due to corticosteroids can arise in any patient. Thus, minimizing corticosteroid use aids in reducing such complications.

Mombaerts et al. describe in detail the efficacy and safety of debulking surgery for corticosteroid-resistant dacryoadenitis; their study was not limited to IgG4-related cases [23]. They found that 80% of patients did not experience disease recurrence after surgery, indicating the procedure’s effectiveness. However, the remaining 20% required additional interventions, such as orbital radiation or rituximab treatment. Although approximately half the patients experienced mild dry eyes as a side effect, the dry eyes were generally manageable with administration of eye drops. Thus, similar to the current study, the overall safety of debulking surgery was demonstrated.

A critical surgical consideration was preserving the palpebral lobe of the lacrimal gland. This approach is grounded in the anatomical and functional significance of the palpebral lobe, which is crucial for preventing postoperative severe dry eyes [14, 24]. Removal of the palpebral lobe can damage the penetrating secretory ducts of the orbital lobe, thus resulting in severe secretion deficiency. In contrast, removal of only the orbital lobe does not result in severe dry eyes because the remaining palpebral lobe works well, even though lacrimal secretion is, to some extent, reduced [25].

This study had several limitations. First, as a retrospective study with a limited patient sample, larger prospective studies are needed for definitive conclusions. Second, the different corticosteroid regimens between groups (routine in biopsy vs. only for relapse in debulking) complicate direct comparisons. Although the lower relapse rate in the debulking group suggests reduced corticosteroid need, this interpretation requires caution due to differing protocols and potential confounders. Third, corticosteroid administration may also be influenced by extraorbital manifestations of IgG4-RD like single case with concurrent sphenoid lesion in debulking group, potentially leading to misinterpretation of treatment effects in the lacrimal gland lesion. In either group, no new extraorbital lesions affecting corticosteroid therapy were identified during the follow-up period, although whole-body CT scans were not performed to avoid unnecessary radiation exposure. Fourth, relapse was defined by subjective findings, as postoperative imaging was not routinely performed to avoid invasiveness. Lastly, mean follow-up periods differed significantly between groups due to timing-dependent decisions of surgical strategies.

In summary, our findings indicate that debulking surgery is a viable and effective method for managing IgG4-ROD, potentially lowering recurrence and dependence on corticosteroids. Moreover, this study highlights the importance of employing the correct surgical techniques to effectively control the risk of postoperative dry eyes.

## Figures and Tables

**Fig. 3:**
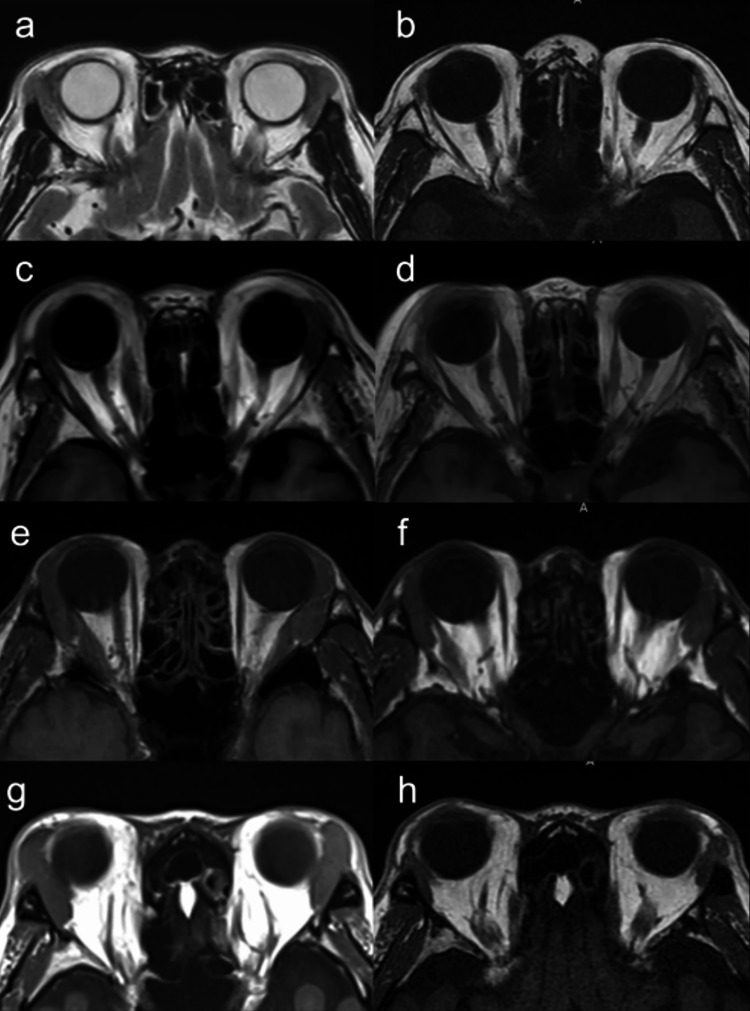
Pre- and postoperative images in the biopsy group (Set 1 of 2). **a**, **c**, **e**, **g** Preoperative images. **b**, **d**, **f**, **h** Postoperative images corresponding to **a**, **c**, **e**, **g**, respectively

**Fig. 4:**
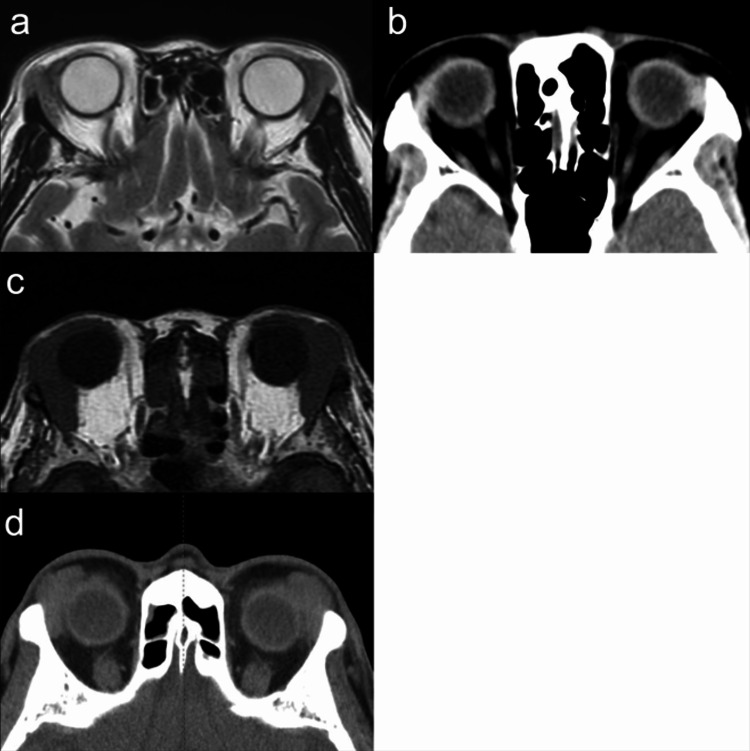
Pre- and postoperative images in the biopsy group (Set 1 of 2). **a**, **c** Preoperative MRI. **d** Preoperative CT. **b** Postoperative CT corresponding to **a**

**Fig. 5:**
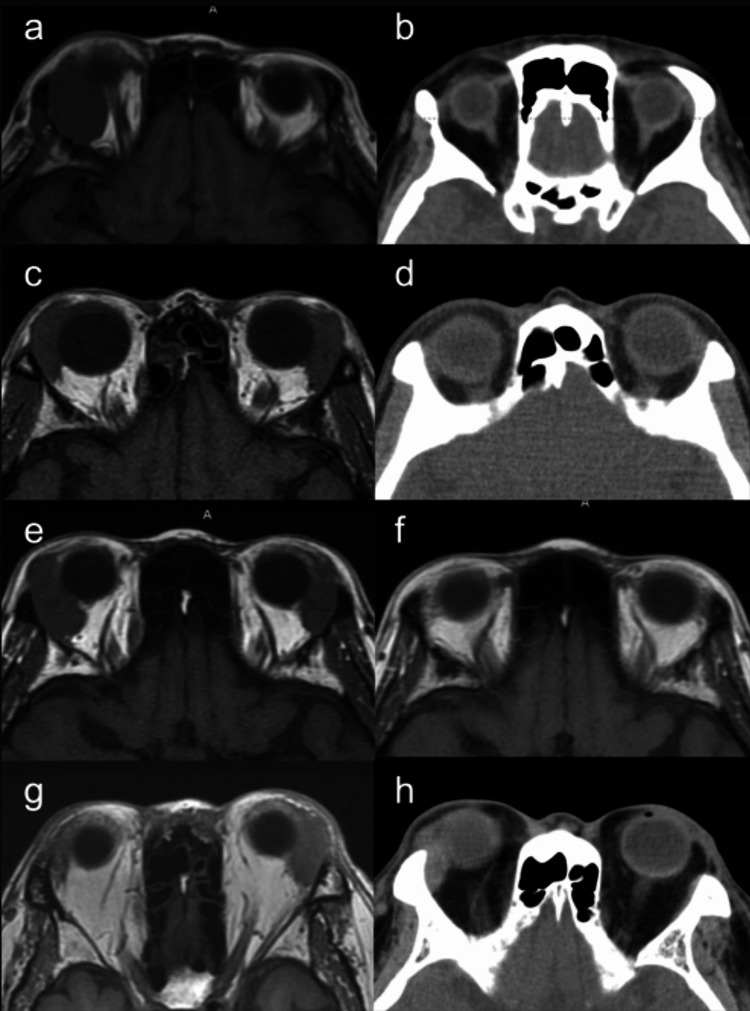
Pre- and postoperative images in the debulking group (Set 1 of 2). **a**, **c**, **e**, **g** Preoperative MRI. f Postoperative MRI and **b**, **d**, **h** Postoperative CT corresponding to **e**, **a**, **c**, **g**, respectively

**Fig. 6:**
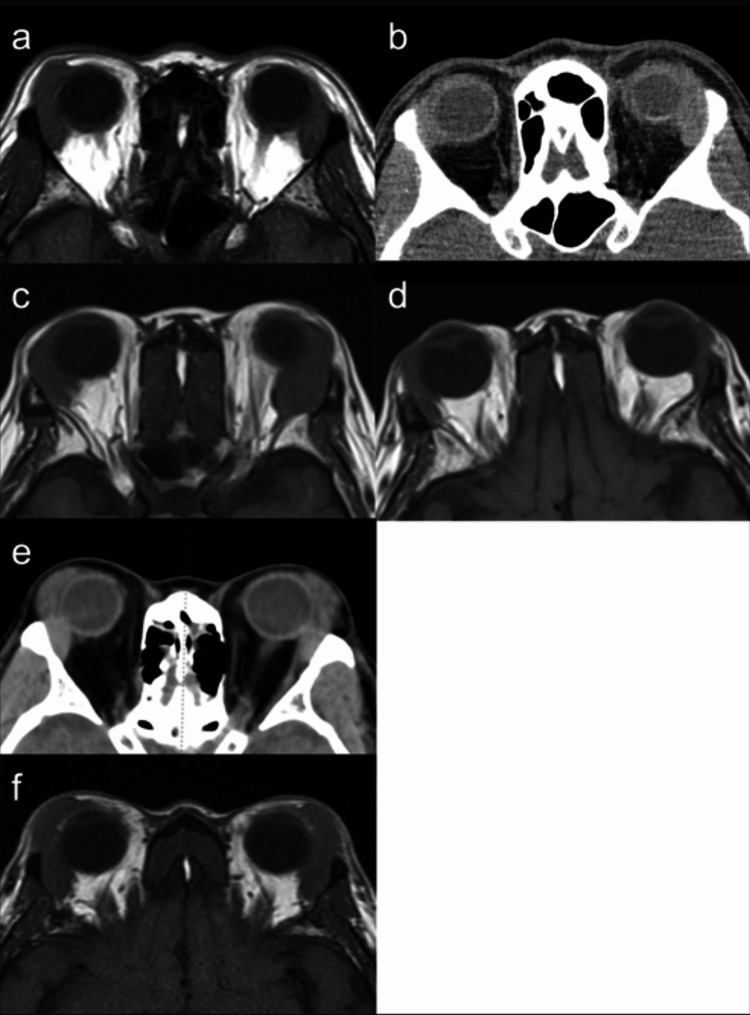
Pre- and postoperative images in the debulking group (Set 2 of 2). **a**, **c**, **f** Preoperative MRI. **e** Preoperative CT. **d** Postoperative MRI and **b** Postoperative CT corresponding to **c** and **a**, respectively

## Appendix

See Figs. 3, 4, 5 and 6.
